# Supplementary material for: Assessing the virulence of Cryptococcus neoformans causing meningitis in HIV infected and uninfected patients in Vietnam
Source: Med Mycol. 2020 Mar 20;58(8):1149–61. doi: 10.1093/mmy/myaa013 (PMC7657091; doi:10.1093/mmy/myaa013)
Supplement: myaa013_Supplemental_File [file myaa013_supplemental_file.docx]

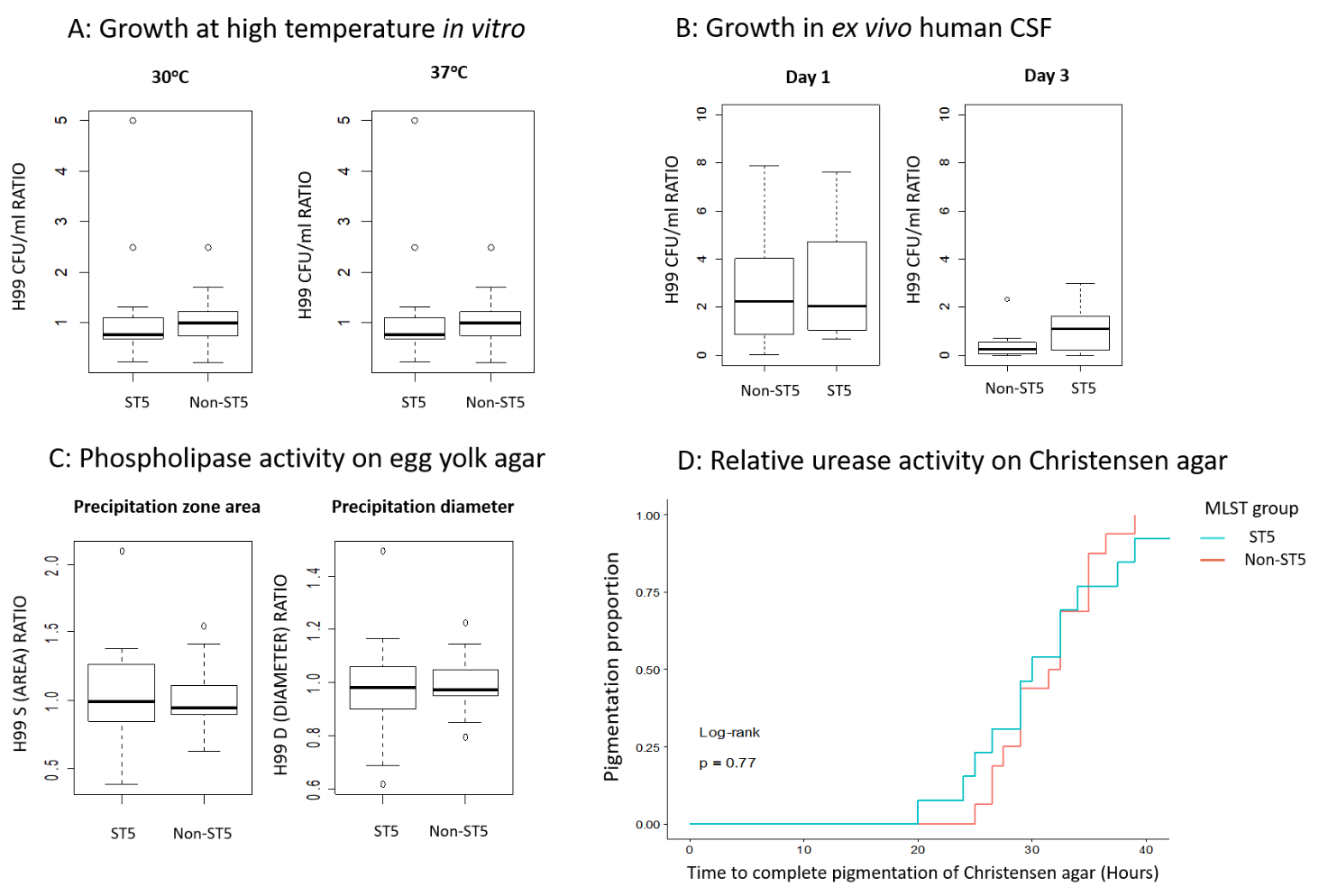

**FIGURE S1. Phenotyping of *C. neoformans* var. *grubii* isolates from Vietnam (ST5: n=15 and non-ST5: n=15).** Data were obtained from 30 isolates (15 ST5 and 15 non-ST5) in 3 different experiment batches, with 3 technical replicates of each isolate per batch. Data are expressed as the ratio between measurements of the test isolates and that of H99. Boxplots (Tukey’s method) describe the median and interquartile range (panel A-C). For assessment of urease activity (panel D), we used the time to complete color change of the agar plate as an indirect measure of extracellular urease activity since all *C. neoformans* var. *grubii* isolates in our collection were positive for urease activity. Each isolate were tested in triplicate and monitored in real time by live-imaging. No significant differences in any virulence-associated phenotypes were observed between the two MLST groups.


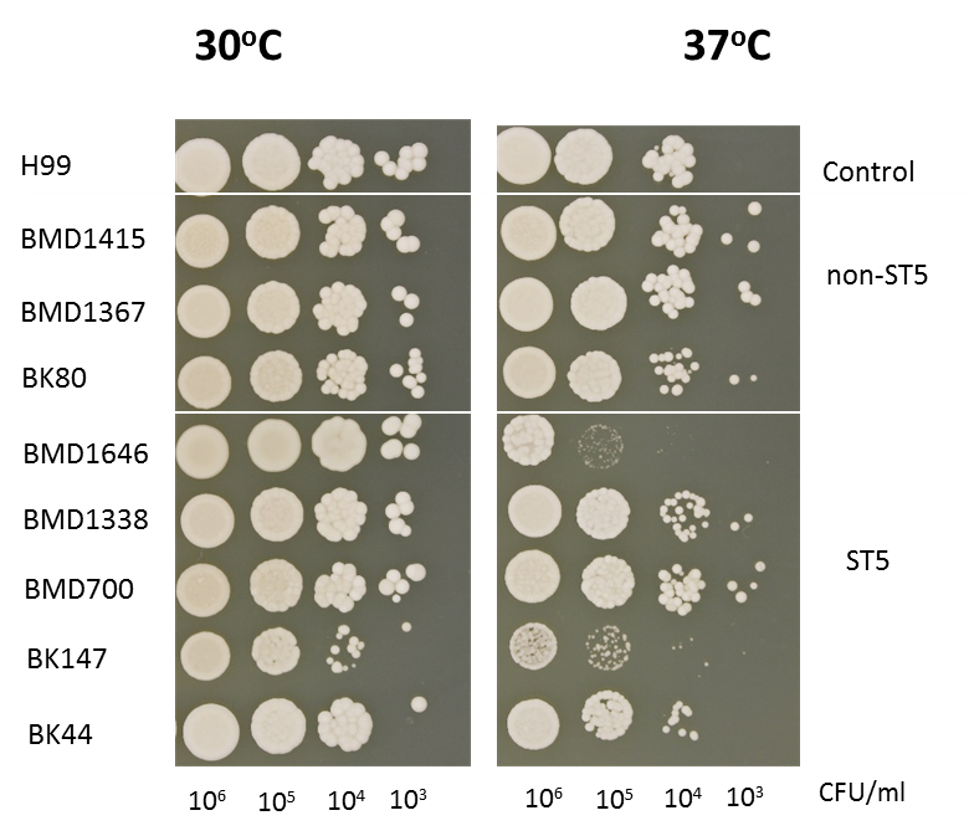


**FIGURE S2.** **Growth at different temperature of 8 *C. neoformans* (five ST5 and three non-ST5) isolates from Vietnamese patients used in the murine infection experiments**. All strains expressed similar growth at 30^o^C. Two ST5 isolates (BMD1646 and BK147) displayed diminished growth at 37^o^C compared to other test isolates.

**
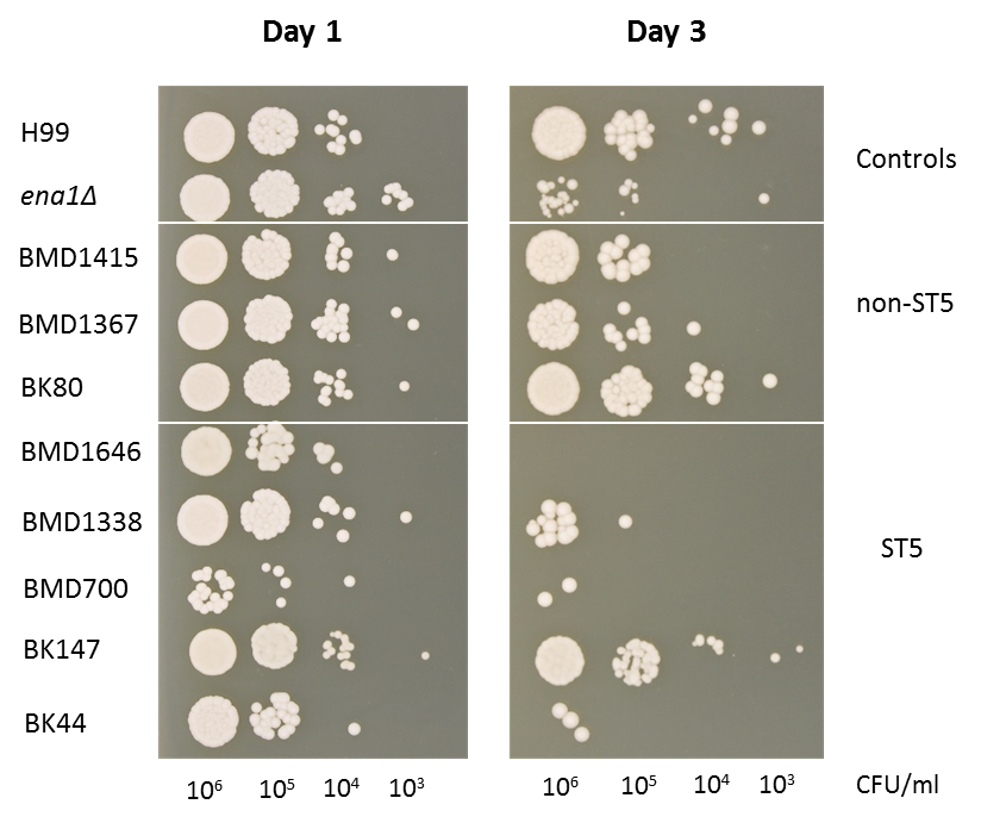
**

**FIGURE S3**. ***Ex vivo* growth in human cerebrospinal fluid (CSF) of 8 clinical *C. neoformans* isolates from Vietnamese patients that were tested for virulence in mice, representing both ST5 (n = 5) and non-ST5 (n = 3) genotypes**. The same inoculum of yeasts were inoculated in pooled human CSF and incubated at 37^o^C. The inoculated CSF was serially diluted and plated on YPD agar at day 1 and day 3 post-inoculation. The wild-type H99 strain and the H99-derived mutant *Δena1*, lacking a cation ATPase transporter which results in decreased viability in human CSF and within macrophages, were included as controls. An apparent difference in growth between ST5 and non-ST5 isolates could be observed at day 3.


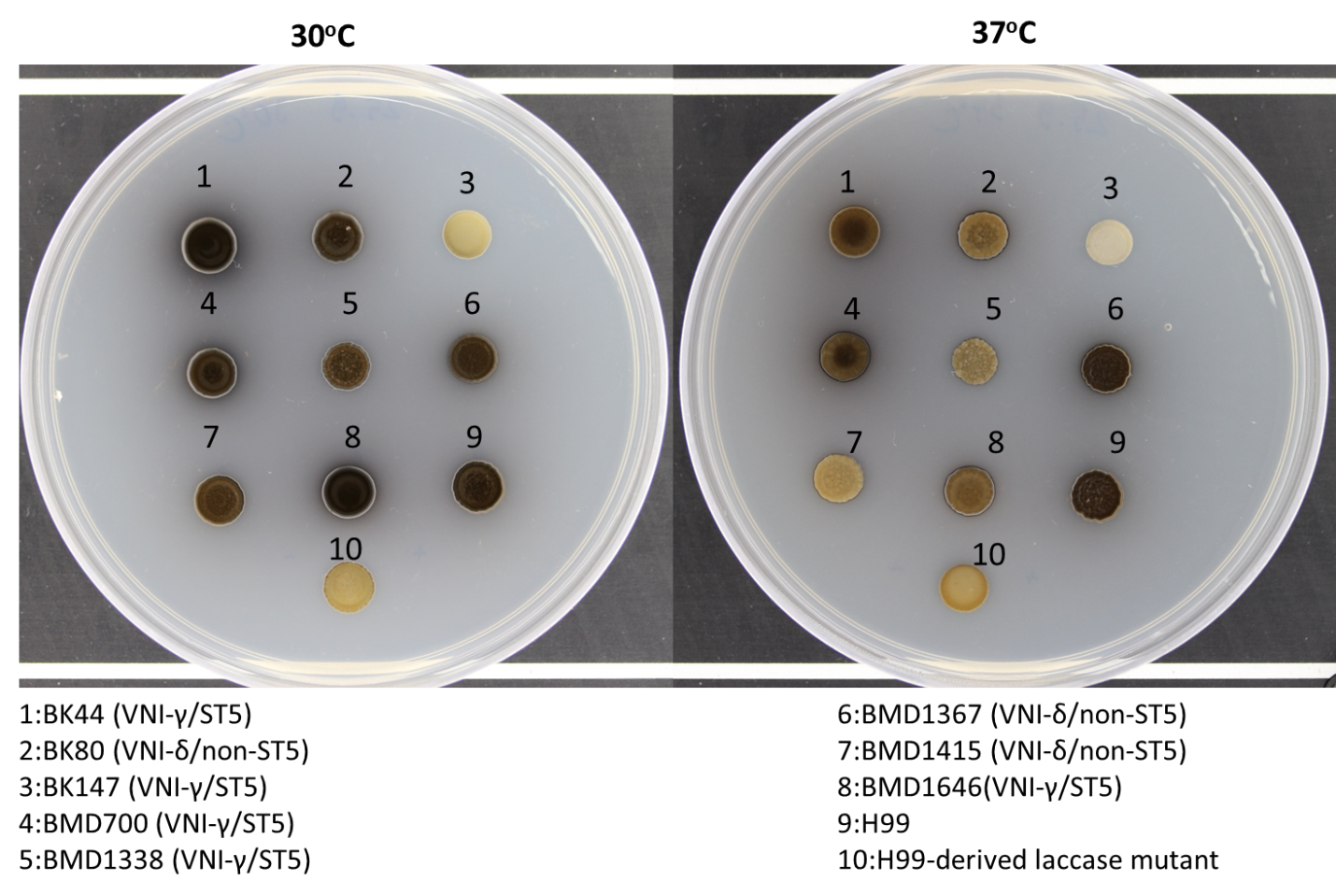


**FIGURE** **S4.** **Melanin production on L-DOPA medium of eight clinical *C. neoformans* isolates from Vietnamese patients representing both S5 (n =5) and non-ST5 (n = 3) genotypes that were tested for virulence in mice**. For each isolates, 10μl of 10^6^ cell suspension was inoculated on L-DOPA agar and incubated in the dark at 30^o^C and 37^o^C. No clear genotype-specific patterns of melanization were observed at either 30^o^C or 37^o^C. The five ST5 strains displayed marked variation in the degree of pigment production, from mildly melanized (BK147) to highly melanized (BK44 and BMD1646).

**FIGURE S5**: **Histopathological scores across 4 categories of tissue damage (Inflammation, necrosis, hemorrhage and edema).** Thin sections of paraffin-embedded lung specimens from infected mice were stained using the Periodic Acid Schiff (PAS) method. Specimens were assessed by an independent pathologist who was blind to infecting isolate and in randomized order. Scores ranged from 0 (no changes) to 10 (severe changes), corresponding to the severity of pathology in in each category as per the Duke Veterinary Diagnostic Laboratory protocol (Division of Laboratory Animal Resources).
